# Bacterial programming of host responses: coordination between type I interferon and cell death

**DOI:** 10.3389/fmicb.2014.00545

**Published:** 2014-10-28

**Authors:** Miqdad O. Dhariwala, Deborah M. Anderson

**Affiliations:** Department of Veterinary Pathobiology, University of MissouriColumbia, MO, USA

**Keywords:** *Yersinia*, plague, *Francisella*, *Salmonella*, *Listeria*, type I interferon, cell death, bacterial infection

## Abstract

During mammalian infection, bacteria induce cell death from an extracellular or intracellular niche that can protect or hurt the host. Data is accumulating that associate type I interferon (IFN) signaling activated by intracellular bacteria with programmed death of immune effector cells and enhanced virulence. Multiple pathways leading to IFN-dependent host cell death have been described, and in some cases it is becoming clear how these mechanisms contribute to virulence. Yet common mechanisms of IFN-enhanced bacterial pathogenesis are not obvious and no specific interferon stimulated genes have yet been identified that cause sensitivity to pathogen-induced cell death. In this review, we will summarize some bacterial infections caused by facultative intracellular pathogens and what is known about how type I IFN signaling may promote the replication of extracellular bacteria rather than stimulate protection. Each of these pathogens can survive phagocytosis but their intracellular life cycles are very different, they express distinct virulence factors and trigger different pathways of immune activation and crosstalk. These differences likely lead to widely varying amounts of type I IFN expression and a different inflammatory environment, but these may not be important to the pathologic effects on the host. Instead, each pathogen induces programmed cell death of key immune cells that have been sensitized by the activation of the type I IFN response. We will discuss how IFN-dependent host cell death may increase host susceptibility and try to understand common pathways of pathogenesis that lead to IFN-enhanced bacterial virulence.

## INTRODUCTION

Type I interferon (IFN) is a major component of the mammalian innate immune system, especially important for defense against viral infection (Stark et al., 1998). Nearly all cells in the body express the type I IFN receptor (IFNAR), making this potent anti-viral response capable of protecting every type of cell. Against bacterial infection, type I IFN can activate inflammatory responses that protect the host, but can also lead to hyper-inflammatory responses and programmed cell death which can hurt the host (Decker et al., 2005). In addition, type I IFN induced during viral infection can lead to increased apoptosis of granulocytes which can prevent clearance of a super-infection caused by Gram-positive or Gram-negative bacterial pathogens (Navarini et al., 2006).

Interferon-β is typically induced following detection of pathogen associated molecular patterns (PAMPs) by membrane-bound or cytoplasmic pattern recognition receptors (PRRs; Takeuchi and Akira, 2010). Expression of type I IFN is regulated at the transcriptional level, with binding sites for multiple activators in *Ifn* promoters. Membrane or cytoplasmic PRRs in the host cell signal through adaptor proteins to activate interferon regulatory transcription factors (IRFs), such as IRF-1, 3, 5, or 7. Phosphorylated IRF migrates to the nucleus, and cooperates with NF-κB and other co-activators to form an enhanceosome that binds the *Ifnβ* promoter and activates transcription (Panne et al., 2007). Secreted IFN-β binds to IFNAR which results in the activation of the JAK-STAT pathway leading to the formation of the interferon-stimulated gene factor 3 (ISGF3) complex (Ivashkiv and Donlin, 2014). This complex translocates to the nucleus and can initiate the transcription of interferon-stimulated genes (ISGs) via their 5′ enhancer elements known as Interferon Stimulated Response Elements (ISREs). These ISGs encode *Ifnβ*, pro- and anti-inflammatory cytokines, activators or inhibitors of programmed cell death, and numerous anti-viral proteins (Sato et al., 1998; de Veer et al., 2001; Schoggins and Rice, 2011).

Nucleic acids, secondary messengers, cell wall, or membrane fragments from bacteria activate expression of IFN-β following its detection by phagosomal or cytoplasmic PRRs (Takeuchi and Akira, 2010; Woodward et al., 2010; Jin et al., 2011a; Parvatiyar et al., 2012). Many pathogenic bacteria survive phagocytosis and may even grow in the intracellular compartment. When intracellular PRRs are activated, a downstream type I IFN response may include increased pro-inflammatory cytokine expression, down-regulation of cytokine receptors, or the sensitizing of key immune cells to undergo programmed cell death. Increasing evidence associates IFN-dependent host cell death during bacterial infection with increased susceptibility to disease. It is clear that the factors that determine the outcome of IFN signaling are complex and influenced by cell-and tissue-specific host-pathogen interactions. In this review, we will discuss type I IFN-dependent sensitization of immune cells to programmed cell death during bacterial infection, the host-pathogen interactions that might enhance this outcome and how it might contribute to disease.

### IFN-DEPENDENT DEPLETION OF IMMUNE EFFECTOR CELLS CRIPPLES HOST DEFENSE

*Yersinia pestis* is a recently evolved vector borne pathogen that causes the lethal diseases bubonic, septicemic, and pneumonic plague (Pollitzer, 1954). All three forms lead to systemic disease and after the infection eliminates virtually all of the phagocytic cells, extracellular bacterial growth is uncontrolled (Heine et al., 2013). When mammals, including humans, inhale *Y. pestis* aerosols, primary pneumonic plague develops in a short period, resulting in a deadly bronchopneumonia that becomes untreatable shortly after symptoms present (Butler, 2013). Neutrophil recruitment and function is critical for host defense against *Y. pestis* infection as well as antibody-mediated protection (Laws et al., 2010; Eisele et al., 2011).

Evasion of the innate immune system by *Y. pestis* is driven by two dominant virulence mechanisms: tetraacylated LPS and a type 3 secretion system (T3SS). Thermal control of acetylases causes hypoacetylation of lipid A at the mammalian body temperature resulting in predominantly the tetraacylated form during infection (Kawahara et al., 2002; Rebeil et al., 2006). Tetraacylated LPS does not stimulate toll-like receptor 4 (TLR-4) and may have anti-inflammatory properties that limit the activation of immune cells by extracellular bacteria (Montminy et al., 2006; Valdimer et al., 2012).

Upon intimate contact with a host cell, the T3SS spans the inner and outer membranes and a translocation pore is formed in the host cell plasma membrane but detection of this pore by the host inflammasome is blocked by the bacterial protein YopK (Cornelis, 2006; Brodsky et al., 2010). YopK is one of seven effector proteins of the T3SS, collectively referred to as *Yersinia* Outer Proteins (Yops), that are transported into the cytoplasm of the host cell where their combined action disrupts signaling pathways, reduces phagocytosis, halts the expression of pro-inflammatory cytokines, and induces programmed cell death through multiple mechanisms (Raymond et al., 2013). This action stalls the inflammatory response, creating an anti-inflammatory environment that is permissive for bacterial growth (Price et al., 2012). Substantial evidence suggests that extracellular bacteria preferentially target macrophages and neutrophils and cause their depletion as the infection progresses (Marketon et al., 2005; Maldonado-Arocho et al., 2013; Pechous et al., 2013).

YopJ is a T3SS effector protein with deubiquitinase and acetylase activity that prevents activation of NF-κB, MAP kinase kinase, and IRF-3, as well as other proteins in the host causing suppression of pro-inflammatory cytokine expression (Monack et al., 1997; Palmer et al., 1998; Orth et al., 1999; Zhou et al., 2005; Sweet et al., 2007). Suppression of NF-κB and activation of RIP1 by YopJ leads to the initiation of apoptosis and pyroptosis, respectively (Monack et al., 1997; Zheng et al., 2011; Philip et al., 2014). The amount of YopJ injected, host cell type and the action of other Yops such as YopK influence the amount of cell death that is caused by YopJ and, although it appears that evolution is favoring reduced secretion of YopJ, this protein and its proper regulation are important to *Yersinia* virulence (Holmstrom et al., 1995; Lemaitre et al., 2006; Brodsky and Medzhitov, 2008; Zauberman et al., 2009; Brodsky et al., 2010; Peters et al., 2013).

The combined evasion provided by YopJ and the tetraacylated lipid A leads to immune suppression that delays neutrophil recruitment allowing for establishment of bacterial colonies in susceptible tissues. Type I IFN expression can be detected early during infection when other pro-inflammatory cytokines are suppressed (Patel et al., 2012). Nevertheless, the absence of type I IFN signaling does not alter the expression of pro-inflammatory cytokines including those harboring ISREs. Overall the data suggest that the immune suppressive environment established during the early stages of *Y. pestis* infection does not prevent expression of IFN-β but nevertheless, at least some ISGs are suppressed.

If a macrophage succeeds in taking up *Y. pestis* before it is injected by the T3SS, the bacteria can remain viable inside a membrane-enclosed compartment known as the *Yersinia* containing vacuole (YCV) where the T3SS functions poorly (Zhang et al., 2011). Intracellular survival requires bacterial stress response pathways which presumably allow the bacteria to adjust to an adverse, nutrient-limiting environment (Oyston et al., 2000; Grabenstein et al., 2004). Intracellular *Y. pestis* eventually lyse the cell and once extracellular, the bacteria appear to have acquired increased resistance to phagocytosis and killing by neutrophils (Ke et al., 2013). *Y. pestis* mutants that are unable to survive in activated macrophages were less virulent in murine plague models suggesting the intracellular life cycle is a biologically relevant process that contributes to the success of infection (Oyston et al., 2000).

Host pathogen interactions that occur as a result of the intracellular life cycle of *Yersinia* are largely uncharacterized. Although extracellular bacteria effectively suppress the expression of pro-inflammatory cytokines, the host likely detects intracellular *Y. pestis* where an abundance of PRRs can bind nucleic acids as well as surface located PAMPs and, to date, no microbial species have been described that escape detection inside host cells. Recently, expression of the mitochondrial-located adaptor protein MAVS was identified as induced by *Y. pestis* infection of macrophages (Du et al., 2014). Mice lacking MAVS were more resistant to *Y. pestis* infection, suggesting that MAVS could play a role in inducing type I IFN. Together the data support the likelihood that one or more intracellular PRRs are activated by *Yersinia*, causing expression of IFN-β.

**FIGURE 1:**
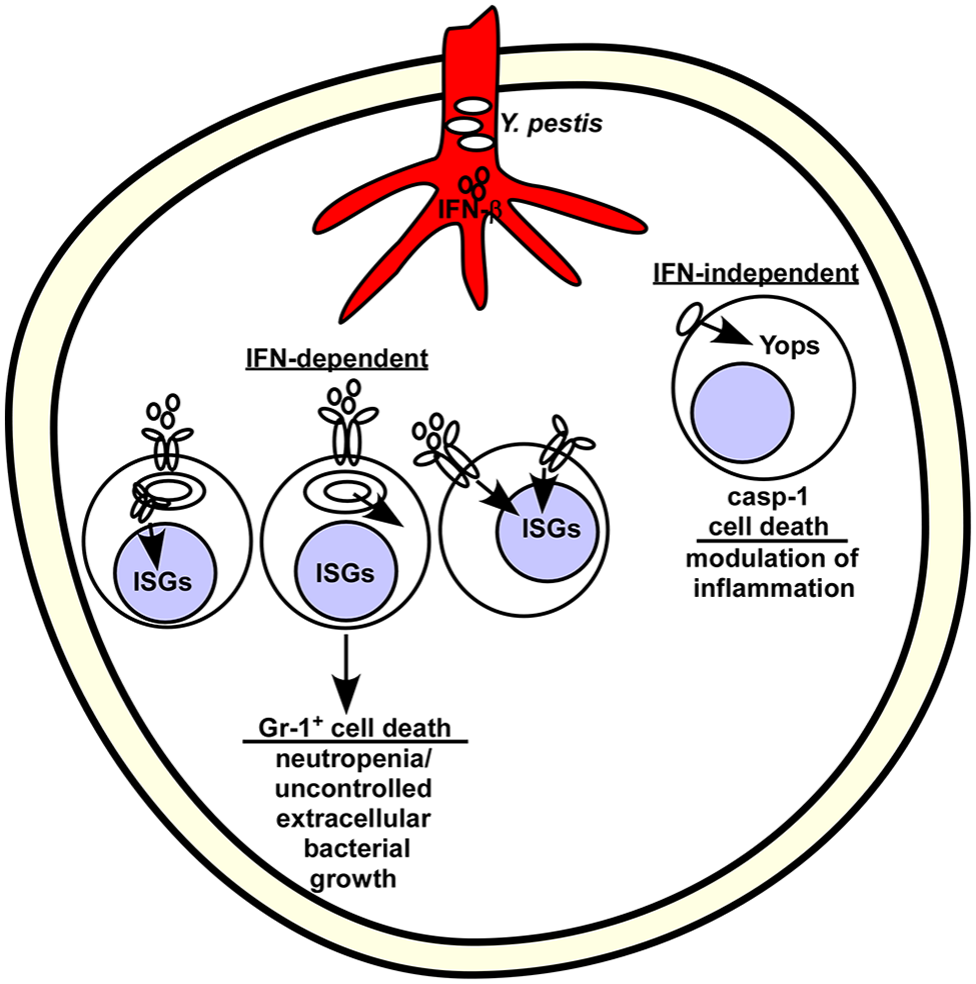
**Model for interferon (IFN)-β stimulated depletion of neutrophils in the bone marrow following *Yersinia pestis* infection.** *Y. pestis* stimulate IFN-β production in the blood shortly after infection. Bacteria likely disseminate from the infection site through the vasculature, where they reach and colonize the bone marrow. Given their tropism for phagocytic cells, *Yersinia* might preferentially interact with Gr-1^+^ cells, which are typically mature neutrophils and monocytes. Extracellular bacteria use the type III secretion system to inject Yops and stimulate caspase-1-dependent, IFN-independent cell death. Intracellular bacteria may stimulate host pathways that combine with IFN to activate cell death (left) or secrete a protein that activates IFN-dependent cell death (middle). Alternatively, the inflammatory signals received by Gr-1^+^ cells may prevent their activation (not shown) or induce cell death (shown).

Pulmonary infection of mice by *Y. pestis* leads to neutropenia that becomes pronounced as the infection progresses (Patel et al., 2012). Mice lacking *Ifnar* were more resistant to lethal disease and this was associated with an increased population of neutrophils in the bone marrow and spleen without detectable changes to the inflammatory response. In contrast, *Ifnar^+/+^* mice had reduced populations of Gr-1^+^ neutrophils in both primary and secondary immune tissues that became more pronounced as the infection progressed. These observations suggest that neutrophil migration is not impacted by type I IFN but more likely it has a direct effect on maturation or viability of this effector population (**Figure 1**). *In vitro*, T3SS^+^ *Yersinia* caused similar levels of cytotoxicity of WT and *Ifnar^-/-^* bone marrow derived macrophages after 5.5 h infection. While this does not rule out the possibility that *Yersinia* infection directly causes IFN-dependent cell death through another mechanism, the data are consistent with an IFN-dependent depletion of neutrophils, perhaps by sensitizing them to undergo cell death *in vivo*.

Interferon-dependent sensitization of immune cells to programmed cell death was among the initial observations of pathology conferred by type I interferon during bacterial infections in the well-characterized model system of another facultative intracellular pathogen *Listeria monocytogenes*. We now know the details of a number of IFN-dependent host responses to *L. monocytogenes*, a pathogen with multiple mechanisms for inducing programmed cell death. Detection of *Listeria* by TLR-2 leads to expression of pro-inflammatory cytokines (McCaffrey et al., 2004; Torres et al., 2004). *L. monocytogenes* can escape this response by invading phagocytic and non-phagocytic cells where it escapes from intracellular vacuoles and grows in the cytoplasm. Detection of nucleotide secreted by bacteria in the cytoplasm is signaled through the adaptor protein STING which leads to the phosphorylation of IRF-3 and expression of IFN-β (Burdette and Vance, 2013).

Multiple mechanisms are believed to contribute to the increased resistance of *Ifnar^-/-^* mice to *L. monocytogenes* infection (Auerbach et al., 2004; Carrero et al., 2004; O’Connell et al., 2004). IFN-dependent susceptibility to infection correlated with increased apoptosis of splenic T cells, not necessarily infected by *Listeria*, which resulted in a reduction of IFN-γ and an increase in IL-10 expression, both of which would suppress macrophage activation and bacterial killing. Further, *Il10^-/-^* mice were also more resistant to *L. monocytogenes* infection, supporting the model whereby IFN enhanced pathogenesis may be affected by changes in IL-10 (Carrero et al., 2006; Biswas et al., 2007). However, the mechanism whereby T cells become sensitized to apoptosis is not yet clear. Paradoxically, IFNAR signaling also causes up-regulation of CD69 in T cells which increased their sensitivity to antigenic stimulation during *Listeria* infection (Feng et al., 2005). Deletion of CD69 blocks protective immunity to *Listeria* even though *Cd69^-/-^* mice produced increased levels of IFN-β and this induced increased levels of T cell apoptosis (Vega-Ramos et al., 2010).

Listeriolysin O (LLO) is a toxin secreted by *L. monocytogenes* that can cause host cell death and is required for bacteria to escape the phagosome and induce type I IFN (Schnupf and Portnoy, 2007). IFN-β sensitizes macrophages to undergo LLO-mediated necrosis, lowering the amount of toxin required to cause cell death *in vitro* (Zwaferink et al., 2008). As macrophages are important for bacterial clearance, this mechanism likely also contributes to disease progression. Thus at least two key immune cells, T cells and macrophages, are sensitized by IFN-β signaling to induce programmed cell death thereby crippling host defense against *Listeria* infection. In addition, IFN-dependent down-regulation of the IFN-γ receptor also decreases activation of infected macrophages, and since IFN-γ is required for bacterial clearance, this likely also contributes to increased susceptibility (Rayamajhi et al., 2010). Overall multiple IFN-dependent changes, some of which involve programmed cell death, may contribute to increased susceptibility of mice to *Listeria* infection.

### IFN-DEPENDENT MODULATION OF INFLAMMASOME ACTIVATION

*Francisella tularensis* causes tularemia, a disease that begins with a very low infectious dose entering via one of a number of routes including inhalation (Dennis et al., 2002; Foley and Nieto, 2010). Inhalation of aerosolized *F. tularensis* leads to bacterial evasion of inflammatory responses and efficient invasion of alveolar macrophages (Hall et al., 2008). *Francisella* escape the phagosome and replicate in the host cytosol, eventually lysing the macrophage. Extracellular bacteria replicate and disseminate systemically likely through the vasculature. Pneumonic tularemia manifests in humans as an interstitial pneumonia that can cause death due to systemic disease and multi-organ failure.

Evasion of innate immune responses by *Francisella* can be attributed to its invasion of host cells, combined with a non-canonical LPS and absence of flagella (Jones et al., 2011). Thus, even though extracellular bacteria are recognized by TLR-2, intracellular bacteria are only weakly immunostimulatory. Once inside macrophages, *Francisella* escape the phagosome and replicate in the cytoplasm, where they eventually cause host cell death. Type A *Francisella* strains, including those that cause disease in humans, carry a duplicated copy of a 30 kb high pathogenicity island that encodes a type 6 secretion system (T6SS) which is required for virulence in the mouse model (Broms et al., 2010; Bröms et al., 2011; Long et al., 2012). Escape from the phagosome, replication in the cytoplasm and host cell death all depend on the T6SS (Lindgren et al., 2013). Like *Listeria*, *Francisella* escape from the phagosome occurs prior to lysosomal fusion and is detected by host PRRs in the cytoplasm which signal through STING to the IRF-3-dependent expression of type I IFN (Jones et al., 2010).

*Francisella tularensis* mutants that are unable to escape the phagosome or that survive poorly in the cytoplasm induce increased expression of IFN-β and increased cytotoxicity due to activation of pyroptosis in macrophages (Peng et al., 2011). Phagosomal escape of *F. tularensis* subspecies *novicida* (*F. novicida*), a type A strain that is virulent in mice but avirulent in humans, and type I IFN signaling activate the absent in melanoma-2 (AIM-2) inflammasome, which leads to cleavage of pro-caspase-1, secretion of IL-1β and host cell death (Henry et al., 2007). *F. novicida* mutants that fail to escape the phagosome were attenuated in the mouse model, suggesting that intracellular survival is necessary for virulence. Similarly, the absence of caspase-1, AIM-2 or the inflammasome adaptor protein ASC all individually caused increased susceptibility to *F. novicida* suggesting that the inflammasome contributes to host defense (Fernandes-Alnemri et al., 2010; Pierini et al., 2013). Paradoxically, IFNAR is required to activate caspase-1 during *F. novicida* infection *in vitro*, but *Ifnar^-/-^* mice were more resistant to pulmonary infection by this strain. This could be explained by type II IFN activation of the inflammasome *in vivo* which has been observed as a compensatory mechanism in the absence of type I IFN. Therefore, *in vivo*, *Ifnar^-/-^* mice are likely not deficient in activating the inflammasome during *F. novicida* infection.

Inflammasome activation by *Francisella* varies depending on cell type as well as bacterial strain which complicate interpretation of the *in vivo* data. Human dendritic cells, for example, induced much less caspase-1 dependent inflammasome activation when infected by *F. tularensis* SchuS4, a type A *Francisella* strain that is fully virulent in humans and mice (Bosio et al., 2007; Ireland et al., 2013). Similar to *Y. pestis* infection, SchuS4 suppresses activation of TLRs and the expression of pro-inflammatory cytokines, but is not able to prevent expression of IFN-β from human dendritic cells (Bauler et al., 2011). In addition, caspase-1 may not play a significant role in host defense against SchuS4 (Dotson et al., 2013). These data suggest that intracellular SchuS4 may escape host cells through a distinct, caspase-1-independent mechanism and the role of type I IFN during infection by this strain is not clear (Lindemann et al., 2011).

Like *Y. pestis*, virulence of *F. novicida* may be enhanced by IFN signaling, as *Ifnar^-/-^* mice were more resistant to pulmonary infection (Henry et al., 2010). Larger populations of IL-17A-producing γδT-cells were found in the spleens of infected *Ifnar^-/-^* mice and this correlated with increased neutrophil recruitment and survival. *In vitro*, IFN-β signaling caused a decrease in *F. novicida*-induced IL-17A expression by γδT-cells suggesting a direct effect of IFN-β on these cells. However, this effect may not extend to the virulent *Francisella* strain SchuS4 which is not only resistant to neutrophil-mediated killing but neutrophils may even contribute to disease caused by this strain (Bosio et al., 2007; Schwartz et al., 2012). Although the role of IFN-β during challenge of mice with *F. tularensis* SchuS4 has not yet been described, pulmonary challenge of *Il17Rα^-/-^* mice by *F. tularensis* SchuS4 did not result in increased survival suggesting IL-17A may not play an important role in this model (Skyberg et al., 2013). It will be interesting to see whether γδT-cells produce IFN-dependent IL-17A during infection by SchuS4 or if this strain induces an alternative response to type I IFN.

### IFN-DEPENDENT ESCAPE FROM HOST CELLS

*Salmonella enterica* is a gastrointestinal pathogen with many serotypes that cause a range of diseases including the lethal typhoid fever (Santos, 2014). *S. enterica* infection begins as an interaction with intestinal M cells and enterocytes which take up bacteria from the small intestine. The bacteria survive in a modified phagosome, also called the *Salmonella* containing vacuole (SCV), and intracellular survival is essential for virulence in the murine and calf models (Libby et al., 1997; García-del Portillo, 2001). Intracellular bacteria eventually cause host cell death, allowing the bacteria access to an extracellular replicative niche where it can grow rapidly. Host cell death appears to be induced by multiple virulence factors that are exported from the SCVs via the type III secretion systems. Occasionally, *Salmonella* gains access to the vasculature and disseminates systemically, resulting in sepsis and multi-organ failure.

*Salmonella* express multiple PAMPs that are recognized by the host, including LPS and flagellin that strongly stimulate TLR-4 and NLRC4, respectively, and induce the expression of type I IFN (Wyant et al., 1999; Rosenberger et al., 2000). In addition, bacterial invasion of non-phagocytic cells results in the recognition of bacterial RNA by the cytosolic sensor RIG-I (Schmolke et al., 2014). Bacterial invasion of macrophages and epithelial cells requires the T3SS encoded on *Salmonella* Pathogenicity Island I (SPI-1; Fàbrega and Vilaa, 2013). Intracellular survival and replication require the T3SS encoded on SPI-2, which is also necessary for virulence in some models (Ochman et al., 1996; Libby et al., 1997; Winter et al., 2010). At least two SPI-2 effector proteins (SpvB and SseL) and one SPI-1 effector protein (SipB) are able to induce host cell death (Browne et al., 2002; Rytkonen et al., 2007). The cell death pathway induced by each of these proteins is distinct from one another, and each may provide an escape mechanism from the intracellular compartment.

**FIGURE 2:**
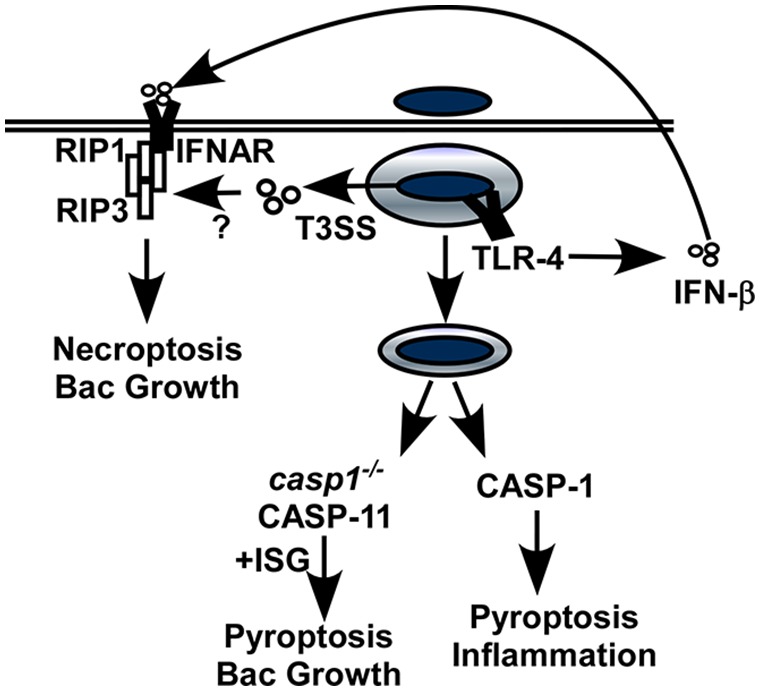
**Interferon-dependent and IFN-independent host cell death caused by *Salmonella.*** Intracellular *Salmonella* remain in a vacuolar compartment where they undergo little, if any replication. The SPI-2 type III secretion system (T3SS) is required for intracellular survival, replication and host cell death. IFNAR promotes necroptosis by forming a complex with RIP1/RIP3, and may also activate the caspase-11 inflammasome under circumstances where caspase-1 is absent. These two pathways favor bacterial replication, presumably because they provide an escape mechanism for the intracellular bacteria. In contrast, IFNAR-independent activation of caspase-1 leads to pyroptosis and inflammation and contributes to clearance of extracellular bacteria.

Mice lacking *Ifnar* were more resistant to *Salmonella* infection with no detectable impact on the expression of pro-inflammatory cytokines (Robinson et al., 2012). Macrophages from *Ifnar^-/-^* mice were resistant to *Salmonella*-induced cell death compared to macrophages from WT mice suggesting IFN signaling may activate host cell death (**Figure 2**). IFN-β induced an interaction between IFNAR and RIP1 in infected cells which promoted RIP1/RIP3-dependent necroptosis. Like *Ifnar^-/-^* mice, *Rip3^-/-^* mice showed improved clearance of *Salmonella* infection suggesting this mechanism contributes to IFN-dependent pathogenesis. While these data provide a direct link between type I IFN, necroptosis and pathogenesis, there are additional mechanisms that are impacted by IFNAR during *Salmonella* infection.

Infection of stationary phase *Salmonella* leads to host cell death *in vitro* through a distinct mechanism involving the SPI-2 T3SS. Through this pathway, *Salmonella* induce the activation of the inflammasome which is enhanced by TLR-4-dependent type I IFN expression (Broz et al., 2012). Yet mice lacking inflammasome caspases 1 and 11 were more sensitive to infection suggesting that inflammasome activation is necessary for host defense against *Salmonella*. This apparent paradox is similar to the observations in the *Francisella* model and therefore may be explained by redundant activation of the inflammasome *in vivo* by type II IFN. Alternatively, specific cells or tissues may mediate susceptibility phenotypes or residual caspase-1 activation in the *Ifnar^-/-^* mice is sufficient for host defense.

*Casp1^-/-^* mice, which express caspase-11 and can activate the inflammasome, were more sensitive to infection than those lacking both caspases that were unable to induce inflammasome activation (Broz et al., 2012). This suggests that caspase-1 and caspase-11 contribute independent rather than redundant functions during infection and that caspase-11-induced cell death may increase disease susceptibility when caspase-1 is absent. Increased resistance of *casp1^+/+^/casp11^-/-^* mice to multiple pathogens has been reported, including *Francisella* and there appears to be a connection with type I IFN signaling and caspase-11-mediated host pathology (Schroder and Tschopp, 2010). Activation of caspase-11 by *Salmonella*-infected macrophages was shown to be dependent on IFNAR, with a role for transcriptional activation of expression pro-caspase-11 as well as an additional function that is not well understood. Overall, these data suggest a second functional link between type I IFN and host cell death through the activation of pyroptosis during *Salmonella* infection. Together, it is clear that even for a single bacterial pathogen, type I IFN sensitizes cells through multiple mechanisms to induce programmed cell death. Escape from the host cell without a protective inflammatory response gives *Salmonella* the opportunity to grow rapidly and disseminate where it can cause severe disease.

## CONCLUDING REMARKS

Studies of IFN-dependent host-pathogen interactions that lead to host cell death have been a focus for the last 10 years of research in bacterial pathogenesis, beginning with the initial observations in the *Listeria* model (**Table 1**). Bacterial secretion systems, often encoded within shared high pathogenicity islands, commonly induce type I IFN expression presumably because the secretion pore and/or the effector proteins are detected by cytoplasmic PRRs. The list of bacterial infections that benefit from IFN-β signaling out-numbers those that are protected by it. Strikingly, the pathogens that benefit from IFN-β signaling are all facultative intracellular bacteria.

**Table 1:** Cell death and type I IFN during bacterial infection of macrophages.

Pathogen	PRR^a^/adaptor	Cell death	Virulence factor^b^	IFNAR^c^	Reference
**IFN-β signaling aids the pathogen**
*Yersinia pestis*
Extracellular Intracellular	None Unknown	Casp-3 RIP1/Casp-8 Casp-1 Necrosis	T3SS Unknown	No Unknown	Zheng et al. (2011), Patel et al. (2012), Weng et al. (2014)
*Francisella tularensis*
Extracellular Intracellular	TLR-2 STING	Casp-1	T6SS	Yes	Henry et al. (2007), Henry et al. (2010), Jones et al. (2010)
*Salmonella typhimurium*
Extracellular Intracellular	TLR-4 TLR-5 GBP NLRP3 NLRC4	RIP1-RIP3 Casp-11	T3SS	Yes	Broz et al. (2010, 2012), Robinson et al. (2012)
*Mycobacterium tuberculosis*
Intracellular	NOD2, STING	Apoptosis Necrosis Casp-1	T7SS	Yes	Chen et al. (2006), Pandey et al. (2009), Manzanillo et al. (2012), Repasy et al. (2013), Dorhoi et al. (2014) Koo et al. (2008), Mishra et al. (2010), Shah et al. (2013)
*Staphylococcus aureus*
Extracellular Intracellular	TLR-2 TLR-9 NOD2	Necrosis Casp-1	HlyA	No Unknown	Mariathasan et al. (2006), Martin et al. (2009), Parker and Prince (2012), Parker et al. (2014)
*Listeria monocytogenes*
Intracellular	NALP3 STING	Casp-1	LLO	Yes	Auerbach et al. (2004), O’Connell et al. (2004), Mariathasan et al. (2006), Jin et al. (2011b)
**IFN-β signaling aids the host**
*Legionella pneumophila*
	RIG-1 NLRC4	Casp-1		Unknown	Amer et al. (2006), Lightfield et al. (2008), Monroe et al. (2009), Nogueira et al. (2009), Plumlee et al. (2009), Lippmann et al. (2011)
*GroupB Streptococcus*
	TLR-7	None reported		n/a	Mancuso et al. (2009), Parker and Prince (2012)
*Streptococcus pneumoniae*
	STING	Necrosis Apoptosis	Ply	Unknown	Weigent et al. (1986), Colino and Snapper (2003), N’Guessan et al. (2005), Sutterwala et al. (2007), Mancuso et al. (2009), Parker et al. (2011)
*Pseudomonas aeruginosa*
	TLR-4 NLRC4	Casp-3 Casp-1	T3SS Unknown	Unknown	Faure et al. (2004), Power et al. (2007), Sutterwala et al. (2007), Carrigan et al. (2010), Parker et al. (2012)

*^a^PRR, pattern recognition receptor.*

*^b^Virulence factor that stimulates cell death.*

*^c^IFNAR = indicates if IFNAR is required for cell death.*

Current sequencing technologies have revealed nearly 3,500 genes that are responsive to IFN-β signaling, including transcription factors and regulators of programmed cell death (de Weerd et al., 2013). Confounding the ability to define ISGs that confer IFN-enhanced susceptibility to infection is the need to identify critical cells whose IFN-dependent response directly contributes to disease. Type I IFN expression specifically in myeloid cells has recently been shown to be critical to clearance of viral infection (Pinto et al., 2014). With the availability of mouse strains that restrict IFNAR expression to specific cells or tissues, it will be possible to study these issues in the bacterial infection models.

Multiple mechanisms of IFN-induced programmed cell death may contribute to bacterial infection in mouse models. There is beginning to be evidence that IFN-enhanced pathogenesis may also occur in humans. For example, virulence of *Staphylococcus aureus* isolated from human patients appears to correlate with increased production of type I IFN (Parker et al., 2014). Given the growing population of immunocompromised people and the confounding effects of co-infections often present in humans, particularly in hospitals, type I IFN may not always be a safe anti-viral treatment option in spite of its undisputed ability to stimulate clearance of viral infections. As we gain in our understanding of how IFN signaling combines with bacterial virulence factors to enhance disease, it may be possible to stimulate the anti-viral effects of type I IFN without placing the patient at risk for bacterial diseases.

## Conflict of Interest Statement

The authors declare that the research was conducted in the absence of any commercial or financial relationships that could be construed as a potential conflict of interest.
